# Substrate-Bound Protein Gradients to Study Haptotaxis

**DOI:** 10.3389/fbioe.2015.00040

**Published:** 2015-03-30

**Authors:** Sébastien G. Ricoult, Timothy E. Kennedy, David Juncker

**Affiliations:** ^1^McGill Program in Neuroengineering, Department of Neurology and Neurosurgery, Montreal Neurological Institute, McGill University, Montreal, QC, Canada; ^2^Genome Quebec Innovation Centre, McGill University, Montréal, QC, Canada; ^3^McGill Program in Neuroengineering, Department of Biomedical Engineering, McGill University, Montreal, QC, Canada

**Keywords:** haptotaxis, substrate-bound gradient, immobilized gradient, digital gradient, reference surface

## Abstract

Cells navigate in response to inhomogeneous distributions of extracellular guidance cues. The cellular and molecular mechanisms underlying migration in response to gradients of chemical cues have been investigated for over a century. Following the introduction of micropipettes and more recently microfluidics for gradient generation, much attention and effort was devoted to study cellular chemotaxis, which is defined as guidance by gradients of chemical cues in solution. Haptotaxis, directional migration in response to gradients of substrate-bound cues, has received comparatively less attention; however, it is increasingly clear that *in vivo* many physiologically relevant guidance proteins – including many secreted cues – are bound to cellular surfaces or incorporated into extracellular matrix and likely function via a haptotactic mechanism. Here, we review the history of haptotaxis. We examine the importance of the reference surface, the surface in contact with the cell that is not covered by the cue, which forms a gradient opposing the gradient of the protein cue and must be considered in experimental designs and interpretation of results. We review and compare microfluidics, contact printing, light patterning, and 3D fabrication to pattern substrate-bound protein gradients *in vitro*. The range of methods to create substrate-bound gradients discussed herein makes possible systematic analyses of haptotactic mechanisms. Furthermore, understanding the fundamental mechanisms underlying cell motility will inform bioengineering approaches to program cell navigation and recover lost function.

## Introduction

Migrating cells rely on extracellular cues to direct motility. Cues can influence cellular responses in a wide variety of ways ranging from differences in surface stiffness to direct protein–protein interactions (Lara Rodriguez and Schneider, 2013). Cytoskeletal rearrangement directed by extracellular cues dictates the navigation of cells through the environment. Cells may encounter gradients of cues, often proteins, where the local distribution of a guidance protein determines the response (Kim and Peyton, 2012), and can induce a directional change in migration. Protein gradients exist in two forms *in vivo*: diffusible, where proteins diffuse away from a source, or substrate-bound, where the cues are fastened either to cell surfaces or to the surrounding extracellular matrix (ECM). It is important to note that the same protein may act both as a diffusible and a surface-bound cue. For example, a secreted protein may diffuse a short distance, become bound, and then perhaps be released, travel further by diffusion, only to be bound again. Alternatively, a transmembrane protein that is initially firmly anchored to a cell membrane may be proteolyzed to release a soluble ectodomain that then becomes a diffusible cue.

Gradients of diffusible proteins have been investigated using a variety of methods (Wu et al., 2013). These have led to a better understanding of chemotropic cell responses and the downstream signal transduction cascades activated. However, some of these same guidance cues likely also function bound to a surface, which critically allows a cell to develop traction force by using the immobilized protein as an anchor in a process called mechanotransduction (Gillespie and Walker, 2001).

The investigation of chemotaxis, the guided navigation of cells in response to gradients of diffusible cues, has a long history, initially being described in the late 1800s (Engelmann, 1881; Pfeffer, 1884; Jennings, 1906). Particularly, well-studied examples include the migration of *E. coli* bacteria toward food sources (e.g., glucose) (Adler, 1966); the common slime mold dictyostelium, which responds to secreted cAMP gradients to direct growth (Gerisch et al., 1975); and lymphocytes, which respond to chemokine gradients to locate a site of immune response (Schall et al., 1990). Based on observations of lymphocyte navigation, Santiago Ramón y Cajal predicted in 1892 that extending axons navigate in response to gradients of secreted guidance cues (Sotelo, 2002).

The earliest studies of cells migrating in culture quickly provided evidence for the significance of the substrate. For example, classic studies by Ross Grainville Harrison used filaments derived from spider webs to show that cells require a solid substrate to migrate (Harrison, 1914). Extending these findings, Paul Weiss developed the principal of contact guidance, arguing that migrating cells and axons are directed by substrate topography during embryonic development (Weiss, 1934). However, it was not until 1965 that Carter (1965) defined the word haptotaxis, differentiating chemotactic guidance in response to a soluble cue from haptotactic migration on a substrate-bound cue. In this seminal study, Carter formed gradients of palladium by using a wire as a shadow mask to scatter a gradient of evaporated metal onto acetate-coated glass. Cells adhered more strongly to the palladium, and directed cell migration was observed from the acetate up the metal gradient. The mechanism was termed haptotaxis (from the Greek: “haptein” to fasten and “taxis” arrangement) to reflect that the cells were navigating in response to the relative strength of the adhesive contacts made with the substrate (Carter, 1965, 1967). Carter suggested that all cell movements within a tissue could be considered haptotactic, including movement traditionally considered to be chemotactic, speculating that the mechanism of action of chemotactic gradients arose from the adsorption of the cue to a surface to form a haptotactic gradient. Such claims, that haptotaxis might underlie all cell movements, not surprisingly, generated controversy, and drew counter arguments from those studying chemotaxis (Keller et al., 1979). Despite numerous studies over the last 35 years that have refined the terminology and included such terms as haptokinesis to differentiate between directed and random navigation (Schumann et al., 2010), this basic debate continues. In many cases, soluble cues may bind to surfaces and many studies of chemotaxis do not explicitly state or investigate possible contributions of haptotaxis. An additional source of confusion is the possible contribution of the substrate, the reference surface (RS), on which the protein cue is patterned. The formation of a protein gradient with gradually increasing surface coverage generally implies an opposing gradient of the RS, which is discussed in detail below.

Gradient geometry varies greatly *in vivo*, but exact characterization has been challenging and is essentially limited to findings from immunohistochemical analyses and assumptions derived from computational modeling. For instance, gradients are expected to have a dynamic range that spans three to four orders in magnitude (OM), and while overall they may increase from one extremity to the other, locally the complex 3D structure of the environment may result in non-monotonic profiles with local changes in gradient slope. This can be observed, for instance, in spinal cord cross-sections stained for the secreted chemotropic guidance protein netrin-1, where the fluorescence increases along the length of the gradient, but includes local fluctuations in fluorescence intensity due to uneven distribution of the protein around cells (Kennedy et al., 2006). Gradient methods need to be versatile to allow parameters such as dynamic range and gradient slope to be defined and manipulated.

In the late twentieth century, molecular biological insights identified multiple families of extracellular molecular cues underlying haptotaxis, clearly demonstrating that the patterns that instruct cell and axon migration during embryogenesis are not merely topographic. Early studies addressed the function of ECM components themselves, demonstrating critical influences on adhesion and migration (Letourneau et al., 1994a,b). Here, we review a wide range of methods to pattern substrate-bound protein gradients to investigate haptotaxis *in vitro*, as well as the experimental and conceptual framework that underpins these studies. Rather than providing a historical perspective into the development of these methods, we limit our discussion to techniques developed over the last three decades, describing their potential applicability to studies investigating mechanisms underlying haptotaxis.

## The Opposing Reference Surface Gradient

**Figure 1:**
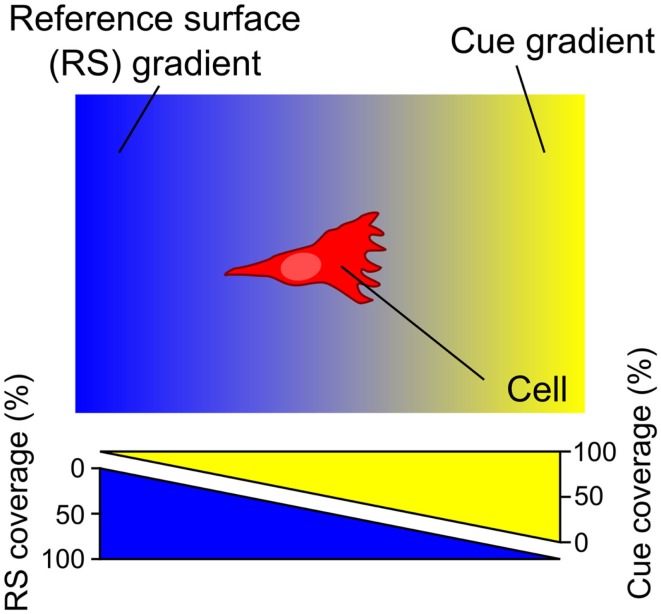
**A surface gradient of a cue (yellow) entails an opposite gradient of the underlying reference surface (RS) (blue)**. The RS, acting relative to the guidance cue, either promotes or inhibits cell adhesion – in either case the RS gradient is expected to modulate the cell response. Consequently, haptotaxis of a cell on a surface-bound gradient is a response to both the gradient of the guidance cue of interest and the opposing RS gradient.

Surface-bound gradients of a guidance cue necessarily entail the presence of an opposite gradient of the underlying RS in the exposed areas of the surface devoid of the immobilized cue of interest (Figure 1).

For all methods that form an immobilized gradient, it is critical to carefully consider the properties of the RS. To investigate its functional significance, the impact of changing the composition of the RS was studied using striped patterns of protein cues (Ricoult et al., 2014b). The gaps (also in the shape of stripes) were completely composed of the RS. A method was developed to tune the affinity of the RS by coating the surface with mixtures of high affinity [i.e., polylysine (PDL or PLL)] and low-affinity [i.e., polyethylene glycol (PEG)], and systematically change the ratio of PDL:PEG from 100:0 (high affinity) to 0:100 (low affinity). It was found that an RS composed of 100% high-affinity PDL, when adjacent to a protein known to promote cell adhesion, functioned to mask the influence of the printed protein, and significantly reduced the navigation speed of cells on the patterned surfaces. Conversely, an RS with 100% low-affinity PEG resulted in increased cell migration on non-guidance proteins, such as stripes of immunoglobulin (IgG).

In the context of a gradient, a high-affinity RS promotes migration in the direction opposite to the cue, while a low-affinity RS promotes migration in the same direction as the cue. Moreover, a low-affinity RS would inhibit adhesion of the cells at the low end of the gradient of the cue of interest and thus skew the distribution of cells on the gradient. In contrast, if the affinity of the RS is high, cells will strongly adhere, and motility will be reduced maximally at the low end of the gradient where a larger proportion of each cell contacts the RS. This reasoning holds for cases where high affinity is synonymous with strong adhesion, which is generally but not always the case. For example, cells may exhibit a high affinity for a laminin coated surface, but not adhere strongly to it (Calof and Lander, 1991). For cells at the low end of a linear protein gradient, the response to the cue will likely dominate as the relative change in protein density is large (e.g., a change from 1 to 2% cue density may likely be significant for a cell), while the relative change in the density of the RS is insignificant (e.g., changing from 99 to 98% coverage may be imperceptible to cells). Likewise, at the high end of the gradient only a small percentage of the RS will be exposed, and the relative change in RS will exceed the relative change in the cue, and hence may dominate cellular responses if the RS is not carefully chosen. While the relative change of exponential gradients is constant along the length of the gradient, the importance of the RS remains just as critical to cellular responses.

In many studies, it is assumed, often based on little if any evidence, that the RS only exerts a non-specific influence and does not trigger a specific biochemical response, yet this assumptions may not be accurate. For example, it is often taken for granted that PDL, which was used as the high-affinity component of our RS, is a biochemically neutral substrate; however, PDL-coated microspheres promote the differentiation of presynaptic specializations by axons (Lucido et al., 2009). Although the mechanism underlying this response remains unknown, its specificity suggests that PDL may activate downstream signaling pathways. More careful consideration of the RS and a better understanding of the molecular mechanism that may be triggered by cell–RS interactions will help refine the analysis of cell navigation. Depending on the experiment and cell type, it may be necessary to develop specific RSs that minimally confound cell navigation.

The response of a cell to a haptotactic cue in the presence of different RSs enables the creation of a cell-surface affinity curve that facilitates comparison between different cell types and surfaces (Ricoult et al., 2014b). The optimal RS will depend on the guidance cue being investigated, the cell type used, and may also depend on the physical properties of the underlying substrate (e.g., hydrogel vs. rigid surface). By choosing a RS of relatively extreme low or high affinity, it is possible to direct cells to migrate onto or off a guidance cue of interest, demonstrating tremendous potential for experimental artifact if the RS properties are not carefully considered. By carefully tuning the RS to a specific intermediate value, it is possible to evoke a biochemically appropriate response and study cell migration in response to a patterned guidance cue (Ricoult et al., 2014b). As such, the optimization of the RS is critical in studies of haptotaxis. Consistent with this, in studies using digital nanodot gradients described in detail in the Section “Protein Gradients Created by Microcontact and Nanocontact Printing,” directed cell migration was not detected using 100% PDL as the RS, but by optimizing the RS, appropriate cell migration then occurred (Ricoult et al., 2013). In summary, the presence of an RS is inherent in haptotaxis experiments. It is critical to carefully select and tune the RS, and to interpret cellular responses to a gradient of the protein cue of interest simultaneously in the context of the RS gradient.

## Methods to Create Substrate-Bound Protein Gradients

An early method developed to create surface gradients of proteins employed vacuum application through membranes. Since then, a myriad of microfluidic devices, including open microfluidics have been developed. Methods of gradient formation from solution now include inkjet printing, chemical gradient formation to modulate adsorption of proteins, and diffusion from hydrogels. An alternative approach to manipulate the protein concentration is to change the density of microscopic “dots” or lines of proteins, which can be realized using techniques that include micro- and nanocontact printing, dip-pen nanolithography, and colloidal lithography. The use of light to form gradients has been explored using photolithography and direct laser writing. Haptotactic gradients can also be made in 3D by cross-linking guidance cues into hydrogels. Stationary microfluidic gradients can thus be “frozen” into 3D gradients using pH sensitive cues, or photopatterning, or by controlling the mixing of two hydrogels. It is also possible to make 3D gradients by making fiber scaffolds. This myriad collection of techniques developed to generate substrate-bound gradients over the last three decades is reviewed in detail in the following sections.

### Protein doping of porous membranes to create gradients

**Figure 2:**
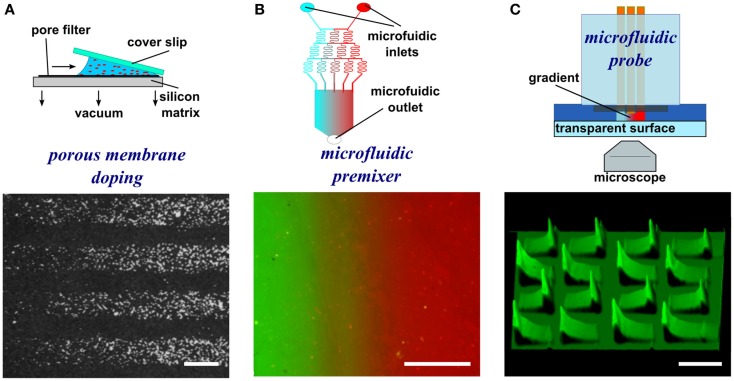
**Schematic representations and experimental results of porous membrane doping and microfluidic methods to form substrate-bound protein gradients**. **(A)** By applying a vacuum, protein solutions can be aspirated through a silicon matrix where the proteins bind. By placing an angled coverslip on top of the solution, a protein gradient can be adsorbed to the matrix. Reprinted with permission from AAAS (Baier and Bonhoeffer, 1992). **(B)** Micrograph image of a microfluidic serial dilutor forming a dilution series that is flowed into a wide chamber forming a gradient across it. With the appropriate surface chemistry, proteins adsorb to the surface, thereby capturing the diffusible gradient on the substrate. **(C)** A microfluidic probe with injection and aspiration apertures serves to create confined microfluidic flows that can pattern gradients (here fluorescence intensity is shown as topography) by continuously changing the writing speed while patterning. Scale bars are **(A,B)** 100 μm and **(C)** 500 μm.

An early method to generate substrate-bound patterns, both stripes and gradients, employed a vacuum and a porous capillary filter (Figure 2A) (Baier and Bonhoeffer, 1992). Using this method, protein in solution is incubated on one side of the porous filter. A vacuum is then applied to pull the solution through the membrane, to which the biomolecules bind as they flow through the porous material. By placing silicon masks with slits on top of the porous filter, the protein flow is limited to the open portion of the mask resulting in patterns of protein adsorption on the surface matching the designs on the silicon mask, such as stripes. By placing a glass coverslip on top of a droplet of solution, the amount of solution available for passage through the membrane changes in a graded fashion. As the vacuum is applied, the solution first becomes depleted on the side of the coverslip with the biggest gap with the surface and move toward the smallest gap. This difference in the amount of protein that interacts with the matrix results in a substrate-bound protein gradient. Despite the efficacy of this approach, other methods with greater control over pattern geometry, a greater range of substrate materials (i.e., not reliant on a porous substrate), and a less complex experimental setup are now available and are discussed below.

### Microfluidic patterning of surface-bound gradients

Microfluidics manipulates minute volumes of liquids at scales ranging from micrometers to millimeters. Microfluidic conditions suppress turbulence and impose laminar flows where mixing occurs solely through diffusion (Whitesides, 2006). Based on the advantages of microfluidics, an assortment of designs that follow closed-channel or open-channel configurations have been developed to create diffusible gradients suitable for chemotaxis studies. Microfluidic gradient generators in general create diffusible gradients that can be imprinted onto a surface by protein adsorption (Jiang et al., 2005). Operation of microfluidic devices may be compromised by the presence of minute obstructions, such as air bubbles that disturb flow and disrupt gradient geometry. It is important to note that the surface concentration profile may not reflect the profile in solution, and as flow continues, the surface may become saturated, or multilayers of proteins may form (Squires et al., 2008). Furthermore, accurate characterization of the immobilized gradients, beyond quantifying fluorescence intensity, which poorly predicts the concentration of immobilized protein, has not been achieved.

#### Microfluidic gradient generators

The simplest microfluidic design is a T-junction where two solutions merge into a perpendicular channel and a gradient is formed by the diffusion of a biomolecule across the laminar flow boundary (Walker et al., 2004). The simplicity of the design limits the size of the gradient, but the implementation of more complicated designs could yield gradients of more complex geometries as well as of greater width. One such example is a microfluidic serial diluter where multiple inlet solutions are repetitively split and merged to create microchannels and the dilution series released in a chamber where it rapidly forms a smooth gradient (Figure 2B) (Jeon et al., 2000). For example, substrate-bound protein gradients of the ECM protein laminin were generated on pre-coated poly-l-lysine substrates using this method. These have been used to investigate the response of rat hippocampal neurons to monotonic surface-bound gradients of laminin (Dertinger et al., 2002), and also the response of *Xenopus* spinal neurons to non-monotonic gradients of laminin in the presence of a diffusible BDNF gradient, the first reported application of a double gradient (Wang et al., 2008). Alternative approaches have been pursued that relatively easily create diffusible gradients (Lee et al., 2009). When adsorbing proteins onto a surface, the high surface-to-volume ratio of microfluidics often leads to depletion of molecules in solution, which can result in the spontaneous formation of substrate-bound gradients. An early demonstration took advantage of this phenomenon and formed gradients along the length of a channel, spanning up to 1.2 OM (Caelen et al., 2000). A more recent example developed channels with a triangular cross-section, thus making use of the higher resistance, reduced flow, and greater depletion at the edges to form gradients (Park et al., 2010). These few examples of microfluidic devices and a great majority of microfluidic devices that yield diffusible gradients in general could be used to pattern substrate-bound gradients by flowing the gradient on the appropriate surface for extended periods. Even though substrate-bound gradients are relatively easy to produce using microfluidic gradient generators, the design of microfluidic chips requires knowledge of microfluidic flow and microfabrication, and as mentioned above, their characterization is inexact.

#### Open microfluidic gradient generators

The closed-channel configuration of most microfluidic devices limits their use to patterning a single continuous gradient, even though non-monotonic gradients can be generated (Figure 2C). To overcome this limitation, microfluidic probes were developed (Juncker et al., 2005) that combine the attributes of microfluidic systems and scanning probes. The probe can be moved over a surface by either controlling the position of the microfluidic probe with micromanipulators or by moving the substrate with a motorized stage. Once an appropriate location is identified, flow can be turned on, and by controlling the flow rates and the lag rate of the probe on the surface, substrate-bound protein gradients can be patterned, such as graded distributions of fluorescently labeled IgG (Juncker et al., 2005; Qasaimeh et al., 2013). By exploiting the attributes of the microfluidic probe, the first gradient array, composed of 16 gradients 500 μm in length, were patterned onto a surface within minutes. Although versatile, one limitation of this method is that operation of such microfluidic probes requires a dedicated microscope as well as a motorized stage to move the MFP.

### Inkjet printed gradients

Inkjet printing is a common non-contact surface patterning approach whereby ink droplets are propelled onto a surface through a nozzle at a desired location. The minimal dot size is dictated by the diameter of the nozzle, which defines the diameter of the droplet. Patterned dots from conventional bio-inkjet printers are ~100 μm in diameter, resulting in a resolution of ~300 μm (Tan et al., 2010). Using a bio-inkjet developed to print protein, a substrate-bound continuous concentration gradient of 1.75 mm in length was generated by altering the number of printed rounds of a fixed concentration between adjacent printed dots of ~75 μm diameter on a glass surface. The gradient was formed by inkjeting between 1 and 20 droplets at the same location; however, a difference in fluorescence intensity of only 0.2 OM was achieved (Campbell et al., 2005), illustrating an important limitation of this method.

### Surface chemistry modifications to control protein adsorption

Rather than directly patterning the proteins as a gradient, it is also possible to first form a gradient in the chemical composition of the surface with different affinities, or “stickiness,” for proteins. For example, a chemical surface gradient may be formed by dip coating a gold-coated surface at a controlled rate in a solution of alkanethiols to yield a gradient of thiol coverage (Morgenthaler et al., 2003). Next, protein solutions are applied to the entire surface, and because proteins will preferentially adsorb to the hydrophobic alkane chains, the chemical gradient will be replicated as a gradient of the protein of interest. Even though the ease and low cost of this approach make it appealing, and variations of the slopes can be produced by controlling the dipping and retraction speeds, complex gradient geometries are challenging to produce. Furthermore, the chemical assembly relies on the presence of an underlying gold surface, which imposes restrictions on the substrates used and complicates cell imaging.

### Gel diffusion to create substrate-bound gradients

**Figure 3:**
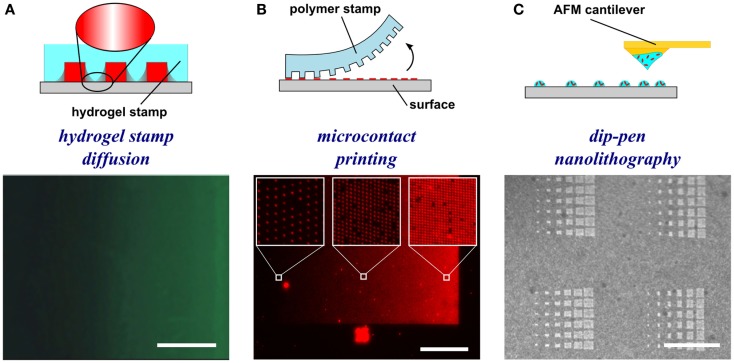
**Schematic representations and experimental results of hydrogel-based diffusion, microcontact printing, and dip-pen nanolithography methods to form substrate-bound protein gradients**. **(A)** Hydrogel stamps in contact with the substrate create closed channels that can be filled with a protein solution. The proteins then diffuse through the hydrogel and adsorb to the surface in a graded distribution. Republished with permission of the *Journal of Neuroscience* (Mai et al., 2009); permission conveyed through Copyright Clearance Center, Inc. **(B)** In microcontact printing, polymer stamps with topography are inked with a protein solution and used to stamp protein in a gradient geometry by limiting the regions of contact between the stamp and the surface. **(C)** Dip-pen nanolithography employs an AFM cantilever inked with a protein solution to transfer localized nanovolumes of protein solution onto the surface in any desired geometry (e.g., dot gradient). Reprinted with permission from AAAS (Huo et al., 2008). Scale bars are **(A)** 100 μm, **(B)** 1 mm, and **(C)** 30 μm.

To generate gradients using gel diffusion, a protein solution is flowed through embedded capillaries in a hydrogel, the proteins diffuse through the gel from the source and adsorb as gradients on the surface (Figure 3A). For instance, agarose stamps with open channels on one of the surfaces are contacted with PLL-coated epoxy coverslips to create closed channels. In one example, netrin-1 or BDNF was then injected into microfluidic channels, allowed to diffuse through the agarose gel, and bound to the surface (Mai et al., 2009). Proteins bound to the surface are more abundant close to the source and became increasingly sparse further away. Gradients are fairly simple to form, but depend on the capacity to bind the diffusible protein to the hydrogel, and control over gradient geometry is very limited.

### Protein gradients created by microcontact and nanocontact printing

**Figure 4:**
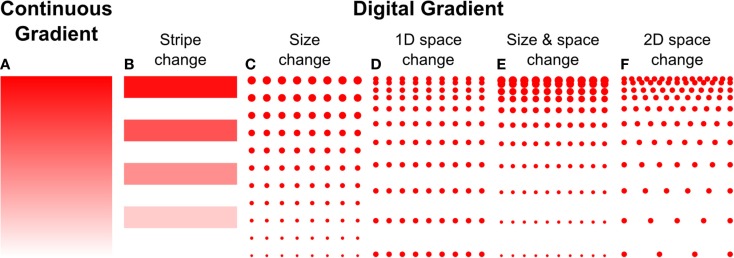
**Haptotaxis gradients can either be continuous or digital**. **(A)** Continuous gradients have constant changes in protein concentration, and usually result from the adsorption of biomolecules from diffusible gradients. Digital gradients can be formed in a number of ways, **(B)** by patterning biomolecules as lines or dots and **(C)** changing their size, **(D)** spacing in 1D, or **(E)** both. **(F)** The spacing can also be changed in 2D.

Microcontact printing employs a polymer stamp with relief to create protein patterns of specific geometry (Figure 3B). The stamp is inked with a protein solution, incubated to allow the protein to adsorb, and subsequently contacted to the surface to transfer the protein only in the contacting areas. A number of different designs have been reported to date that can either be continuous or digital (Baier and Bonhoeffer, 1992; Lang et al., 2008). Continuous gradients can be formed by adsorption of proteins from solution onto a stamp using the methods described in the preceding paragraphs, and then printing the stamp on a substrate (Figure 4A). In continuous gradients, the density of proteins on the surface changes smoothly. Digital gradients on the other hand are formed by patterning proteins as discrete surface-bound “patches” and forming a discontinuous gradient by changing the surface coverage density of the patches. Conventionally, the concentration of proteins in the patches is constant, in many cases saturated with a “monolayer” of proteins, while the patches can be shaped as dots, dashes, or lines. Note that continuous gradients are in fact also digital since, ultimately, individual proteins adsorbed on the surface form discrete “patches,” but the term digital gradient is reserved for gradients that were designed to be discontinuous at a much larger scale ranging from tens of nanometers to millimeters.

A great variety of digital, discontinuous gradients have been made. One conceptually simple approach is to form lines with increasing concentrations by flowing different concentrations of the same molecule through a series of parallel channels on a flat PDMS stamp, resulting in protein adsorption to the stamp. Subsequently, the stamp was used for microcontact printing to transfer the lines onto a flat substrate (Figure 4B) (Lang et al., 2008). A more common approach to form digital gradients is to form patches – a dot or a line – and continuously change the spacing or the size, or both, in the direction of the gradient. Digital gradients with constant spacing and a variable patch size (Figure 4C) were formed by a dashed line with a centrally located node where the length of the dashes is changed based on the distance from the central node (Fricke et al., 2011). The two gradients described above are straightforward to make, but exhibit relatively large surfaces without guidance cue and feature a limited dynamic range. Digital gradients with the spacing of features changed in one direction (Figure 4D) were demonstrated with dots and lines at the microscale (von Philipsborn et al., 2006) and at the nanoscale (Coyer et al., 2007). Coyer et al. also demonstrated gradients with changes in both spacing and feature size (Figure 4E); however, the dynamic range of these gradients was limited to 1.4 OM, and the overall fabrication process remained costly for the nanoscale gradients. Digital nanodot gradients were introduced with the spacing of 200 nm dots changed along two directions (Figure 4F) along with a low-cost patterning method that reduced the cost of individual gradients to a few cents (Ricoult et al., 2013). An advantage of digital gradients is that the local concentration can be read from the dot density, which unambiguously allows defining the density over a large dynamic range. It will be of interest to determine how the size of the nanodots and the protein concentration modulate cellular responses. Whereas prior studies indicated that a feature size of 200 nm was needed to elicit a response (Geiger et al., 2009), no consensus has been reached. The influence of the surface concentration of the protein that makes up the nanodots has not been studied, and a better understanding of the functional significance of these two parameters, in combination with the RS, and how they relate to continuous gradients, will help refine the design of gradient studies in general.

**Figure 5:**
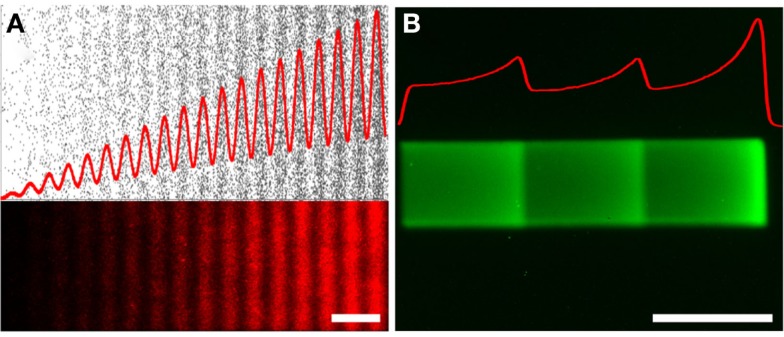
**Non-monotonic haptotaxis gradients**. Non-monotonic protein gradients achieved with **(A)** low-cost lift-off nanocontact printing showing (top) the dots of the digital design (black), and (bottom) the fluorescence image of a red fluorescently labeled IgG of the same non-monotonic DNG. **(B)** Non-monotonic gradients can also be achieved through laser-assisted protein patterning (LAPAP), here illustrated by patterning a fluorescently labeled IgG. The red curves illustrate the density functions of the patterned gradients. Scale bars are **(A,B)** 50 μm.

Using these new designs, the gradients reached an unprecedented dynamic range of 3.85 OM (Ongo et al., 2014), which could more accurately represent the expected dynamic range of gradients *in vivo*. More complex algorithms were also developed to introduce noise into the gradients at the nanoscale by pseudo-randomly distributing dots within a row of constant density and compensating for dot overlap based on the probability of overlap at the given density. Furthermore, noise was also introduced at the microscale by developing gradients where local dot density was fit to non-monotonic functions (Figure 5A). The power of these designs, the relative ease and high throughput of the technique result in a very flexible method. As the resolution and availability of prototyping methods continue to increase (e.g., direct writers and 3D printers) the time and cost associated with the electron beam lithography production of the digital nanodot gradient masters will almost certainly be further minimized. Furthermore, new advances in microcontact printing, such as humidified microcontact printing (Ricoult et al., 2014a), have facilitated the printing of nanopatterns composed of multiple proteins and could lead to the patterning of multiple gradients of different cues.

### Dip-pen nanolithography

In dip-pen nanolithography, cantilevers are employed as reservoirs to pattern surfaces (Figure 3C). By using an agarose solution embedded with protein, biologically active protein can be patterned on high energy, activated surfaces (Senesi et al., 2009). By changing the contact time of the tip with the surface, the humidity level of the environment, and viscosity of the agarose, the dot size can be reduced to 50 nm in diameter. By changing these parameters from dot to dot, an array of protein dots with increasing size can be produced, thereby creating a gradient. Recently, polymer pyramid arrays have been employed in essentially the same way, based on regulating the force applied to control the portion of the pyramid that contacts the surface and therefore the size of the feature (Huo et al., 2008). Furthermore, embedded heaters have been added to polymer pyramid arrays to control polymer expansion under each pyramid to individually manipulate dot size (Brown et al., 2013). Dip-pen lithography provides one of the highest resolutions among the various patterning techniques currently available and recent developments such as low-cost cantilever arrays or individually computer controlled cantilevers have made this method very promising; however, in its current state, the method remains relatively slow and impractical for routine use in most biology laboratories.

### Colloid lithography

**Figure 6:**
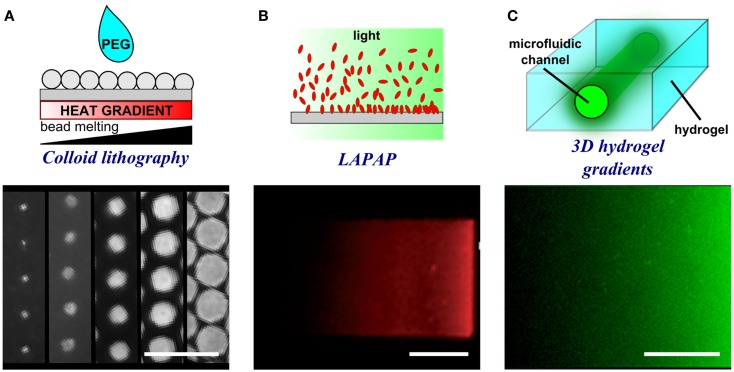
**Schematic representations and experimental results of porous membrane doping and microfluidic methods to form substrate-bound protein gradients**. **(A)** By applying a heat gradient under the surface, microspheres melt and interact with the surface to different extents thereby creating a gradient of dots of different size. Adapted with permission from Taylor et al. (2012). Copyright 2012 American Chemical Society. **(B)** Controlled photobleaching of fluorescently labeled proteins can also be used to increase the reactivity of the proteins with the surface in specific locations and by controlling light intensity or exposure, gradients can be formed. Reproduced in part from Bélisle et al. (2008) with permission of The Royal Society of Chemistry. **(C)** The presence of a microfluidic channel within a hydrogel enables the filling of a point source solution within a 3D environment. The proteins diffuse from the source and create a gradient in a similar fashion to hydrogel stamp diffusion. Reproduced in part from Lienemann et al. (2015) with permission of The Royal Society of Chemistry. Scale bars are **(A)** 20 μm, **(B)** 25 μm, and **(C)** 250 μm.

In colloid lithography, polystyrene spheres in solution are deposited on a surface, and as the solvent evaporates, the spheres form a regular, packed monolayer (Taylor et al., 2012) (Figure 6A). Spheres are then deformed by exposing them to a temperature gradient, which melts the spheres differentially based on the heat applied. By having a heat source to create a temperature gradient on the surface, the extent of sphere melting goes from high, close to the source, to absent, at the most distant position. A PEG silane is then evaporated on the surface and self assembles where the beads do not contact and protect the surface. Spheres are then detached and proteins incubated to fill the gaps previously protected by the beads. The colloid lithography approach yields very large patterned surfaces; however, the limited control of dot placement results in frequent defects. Furthermore, this approach is currently limited to generating linear gradients.

### Block copolymer lithography

In block copolymer nanolithography, polymeric micelles with a polar core and containing metal nanoparticles self-assemble on a surface in an arrangement defined by the architecture of the micelles (Spatz et al., 1999). The polymer portion of the micelles can then be eliminated from the surface by plasma exposure to leave behind patterns of nanoparticles. A dipping machine is then used to change the time of exposure of the surface to the solution. By changing the 2D dot spacing from 55 to 85 nm over 3 mm, it was possible to produce a gradient of ~0.5 OM (Hirschfeld-Warneken et al., 2008). The metal particles were then selectively decorated with thiol-linked peptides, based on the high affinity of thiols for gold surfaces. Block copolymer lithography is an appealing method for nanopatterning, given the ease of the procedure and the commercial availability of a range of micelles; however, the dynamic ranges of the gradients generated remain limited and the capacity of the method to create complex gradients remains unclear.

### Patterning with light

#### Photolithography

An early technique to pattern with light is photolithography, a relatively harsh microfabrication method that uses strong UV light projected through a metal mask to expose specific regions on a surface. The surface may be coated with a photopolymer, or photoresist, that upon exposure to UV light alters the level of polymer crosslinking. Alternatively, selective UV light exposure photolithography has been employed to eradicate specific portions of self-assembled monolayers (SAMs) of thiols terminated with PEG end groups. The eradication of such groups then opens space on the surface for proteins to bind (Hynes and Maurer, 2012). Peptides can also be selectively adsorbed to surfaces coated with thiol SAMs when exposed to light in a process called photoimmobilization. By selectively exposing the surface to light of graded intensity with a photomask, surface-bound peptide gradients can be patterned (Herbert et al., 1997). Photolithography provides a means to pattern very large surfaces in one step; however, the method typically requires a costly clean-room environment. Furthermore, its dependence on light limits resolution to the microscale.

#### Laser-based lithography

In this approach, the fluorescence light source of a microscope is used to either selectively bind or detach biomolecules from a surface. In LAPAP, fluorescently labeled biotin is photobleached, rendering it reactive and causing it to covalently bind to an underlying BSA-coated surface (Figure 6B) (Bélisle et al., 2008, 2013). Streptavidin is then bound to the immobilized biotin and serves as a site to link navigational guidance cues. In contrast, in laser scanning lithography light is used to detach bound molecules from the surface (Slater et al., 2011), for example, to replace detached non-fouling thiols with peptide-binding thiols on a gold surface.

Any desired pattern of high complexity can be designed computationally and sent to a microscope to automatically create the surface-bound patterns (Figure 5B). For instance, by altering the laser intensity and the displacement velocity of the fluorescence focal spot, gradients can be produced (Bélisle et al., 2008, 2013). Notably, this method combines a high level of flexibility with regard to the pattern produced and the capacity to locally coat a substrate with different concentrations of protein. Furthermore, by conducting multiple light exposure and incubation steps, multi-protein patterns can be readily achieved, even generating protein gradients running in opposite directions (Bélisle et al., 2009). Laser-based patterning methods are powerful techniques where intricate designs spanning up to 3.0 OM can be patterned, but the chemistry required to link the protein to the surface is not universal, placing some limits on the proteins that can be used for this type of assembly.

### 3D gradients

Three dimensional (3D) substrates have grown in popularity due to their potential to generate patterns of even greater relevance to mechanisms that occur *in vivo*; however, with the implementation of 3D systems, greater complexity arises and data extraction and analysis becomes more complicated. Nevertheless, a number of methods have been developed to generate gradients in 3D.

#### Hydrogel-based protein gradients

The creation of 3D biomaterials for tissue engineering applications also commonly employs hydrogels (Figure 6C) (Lutolf and Hubbell, 2005). A main attribute of hydrogels is that they are relatively easy to use and their chemistry can be readily designed to include protein binding sites, for example, to capture tagged proteins from solution and integrate them into a PEG-based gel (Lienemann et al., 2015). By forming a soluble gradient in the gel using either a point source diffusion (Lienemann et al., 2015), or a microfluidic gradient generator (Allazetta et al., 2011), proteins can be captured in distributions that reflect the geometry of the soluble gradient. Microfluidics can also be used to create gradients of cross-linked peptides in collagen gels (Sundararaghavan et al., 2011). Alternatively, by electrically manipulating pH within a PEG hydrogel, it is possible to create gradients of gel polymerization based on the distance from the pH electrode (Milleret et al., 2014). Motifs have also been patterned within a hydrogel by integrating photolabile caged biomolecules and using light to selectively release the biomolecules and initiate enzymatic patterning of a PEG gel (Mosiewicz et al., 2013). Gradients presented in hydrogels better mimic the *in vivo* system; however, control over gradient geometry is currently limited, but could be further improved by adapting some of the methods for high resolution 2D patterning to 3D patterning techniques, such as nanocontact printing or dip-pen nanolithography.

#### Spin coating fibrous scaffolds of graded protein content

A popular method to create 3D scaffolds is to spin coat fibers onto a surface. One such material that can be spin coated is hyaluronic acid (HA), an ECM component widespread in mammalian brain (Bignami et al., 1993). Sundararaghavan et al. (2011) altered the conventional method of spin coating by adding a T-channel to the spinneret of the machine, which allowed the two solutions to be mixed at different rates over time. By changing the mixing ratio as the fibers were deposited on the surface, a gradient along the *z* axis could be created within the 3D fibrous scaffold. By mixing either two HA solutions of different modulus or two HA solutions containing different proteins, mechanical and chemical gradients were created that were then used to investigate durotaxis and haptotaxis, respectively (Sundararaghavan and Burdick, 2011). This method of layering spin coated fibers provides a means to achieve more complex 3D gradients; however, the size of the gradients achieved through this method currently greatly exceed that of typical biological gradients and it is unclear whether cells have the capacity to respond to gradients patterned at this scale.

## Conclusions and Outlook

There is substantial incentive to better understand cell navigation, providing further insight into the mechanisms underlying embryonic development and enabling the production of effective biomaterials for regenerative medicine. Multiple proteins known to direct cell and axon migration during development are bound to cell surfaces and the ECM, suggesting that haptotactic mechanisms play a critical role. To better understand haptotaxis, many methods have been developed to create substrate-bound gradients *in vitro*, which are summarized above and in Table 1.

**Table 1:** **Methods for forming substrate-bound protein gradients**.

Method	Throughput (serial → multiplex)	Low-cost	Patterning size	Capacity to adjust the local protein conc.	Resolution (milli → nano)	Dexterity of geometry (fixed/flexible)	Gradient design complexity	Reference
Serial diluters	+	+++	++	+++	Micro	Fixed	+	Dertinger et al. (2002)
Microfluidic probe	+	+	+++	+++	Micro	Flexible	+	Juncker et al. (2005) and Qasaimeh et al. (2013)
Inkjet printing	+	+	+++	+++	Micro	Flexible	++	Campbell et al. (2005)
Vesicle mixing	++	+++	++	+++	Micro	Fixed	+	Kam and Boxer (2000)
Surface modification	+++	+	+++	++	Micro–nano	Fixed	+++	Morgenthaler et al. (2003)
Protein doping of porous membranes	+	++	++	+	Micro	Fixed	+	Baier and Bonhoeffer (1992)
Gel diffusion	++	+++	++	+	Micro	Fixed	+	Mai et al. (2009)
Microcontact/nanocontact printing	+++	+++	++	+	Micro–nano	Fixed	+++	Ricoult et al. (2013)
Dip-pen nanolithography	+	+	+	+	Nano	Flexible	+++	Senesi et al. (2009)
Colloid lithography	+++	+++	+++	+	Nano	N/A	+	Taylor et al. (2012)
Block copolymer lithography	+++	+++	+++	+	Nano	Fixed	+	Hirschfeld-Warneken et al. (2008)
LAPAP	+	+	+	+++	Nano	Flexible	+++	Bélisle et al. (2008, 2013)
Laser scanning lithography	+	+	+	+++	Micro	Flexible	++	Slater et al. (2011)
Photolithography	+++	+	+++	+	Micro	Fixed	++	Herbert et al. (1997)
Polymer scaffolds	+	++	+++	+++	Milli	Flexible	+	Moore et al. (2006) and Sundararaghavan et al. (2011)
3D electrospun gradients	+	+	+++	+++	Micro	Flexible	+	Sundararaghavan and Burdick (2011)
Hydrogels	+	++	+++	+++	Micro	Flexible	+	Mosiewicz et al. (2013)

For all of these assays, it is important to carefully consider the RS. For example, a method was developed to adjust the RS in cell-surface affinity screens that were conducted on stripe patterns (Ricoult et al., 2014b), and to select the condition deemed optimal for nanodot gradient studies (Ricoult et al., 2013). In future studies, the RS should be carefully engineered, tuned, and characterized to allow evaluation of the contribution of the opposing RS gradient to the cell response. Appropriate consideration of the RS will facilitate comparisons with other studies and accelerate understanding of the mechanisms underlying motility.

Gradients patterned using the methods described above can be divided into two classes: continuous and digital, where both have advantages and disadvantages. For instance, continuous gradients can easily be achieved through the diffusion of a protein from a source, but the accurate quantitative characterization of such gradients is challenging and often inexact. Digital gradients are also easily produced, but they are deterministic, and as a result the exact local concentration can be calculated. However, the size of the patterned dots of protein aggregates formed typically remain much larger than the size of individual proteins forming such aggregates in natural gradients, which may influence cellular responses.

The numerous technologies that are now available to form substrate-bound protein gradients provide multiple options to study the mechanisms underlying haptotaxis. Patterning techniques such as LAPAP or dip-pen nanolithography offer the capacity for designs with high resolution, whereas substrate-bound gradients achieved through microfluidic gradients or microcontact printing can rapidly pattern large surfaces at a low cost without need for particularly specialized equipment. Furthermore, using digital nanodot gradients, large arrays of distinct gradients can be patterned onto a same substrate by lift-off nanocontact printing, enabling the evaluation of large numbers of parameters in a single experiment, quantifying their effects, and providing insight into the mechanisms underlying haptotaxis.

Current methods such as LAPAP and low-cost lift-off nanocontact printing enable technically straight-forward patterning of substrate-bound protein gradients with high dynamic ranges (i.e., 3.85 OM), non-monotonic complexity, high resolution, and a composition of multiple proteins. Further developments aim to recreate gradients of high biological relevance in environments that better match those found *in vivo*. By combining protein immobilization with the above-mentioned methods, 3D multi-protein patterns have been achieved, but their resolution remains to be improved to achieve the high resolution possible using methods such as dip-pen nanolithography. Further improvements are focused on developing gradients that will change geometry as the biomaterial ages, for instance, to reflect variations in the gradient geometry that occur as development takes place (Kennedy et al., 2006). Lastly, ongoing studies aim to develop methods to ensure that the conformation of the patterned protein is minimally impacted by the stresses induced by the patterning technique and the surface chemistry. For example, in a landmark study, it was shown that fibronectin adsorbed onto surfaces of distinct wettabilities elicited different ligand–receptor interactions and cell differentiation (García et al., 1999), which was attributed to conformational changes in the fibronectin. The study by García et al., however, did not consider that changing the RS might also have an effect on the cells (Ricoult et al., 2014b). Using the recently developed humidified μCP method (Ricoult et al., 2014a), it is possible to pattern proteins onto a range of RSs with different hydrophobicities and evaluate the cell response. Indeed, it was observed that cells, when in the presence of an appropriate RS, respond to printed proteins through predictable biological pathways, seemingly indistinguishable from the response observed on passively adsorbed protein (Ricoult et al., 2014b). Taken together, these studies highlight that there are many open technical and biological questions related to haptotaxis that remain to be explored. The continued development of methods for forming immobilized gradients on a variety of RS in 2D and 3D will provide better models to study cell biology and benefit many other areas such as tissue engineering.

## Conflict of Interest Statement

The authors declare that the research was conducted in the absence of any commercial or financial relationships that could be construed as a potential conflict of interest.
